# Computed Tomography and Magnetic Resonance Findings in Athletes at Risk for Sudden Cardiac Death

**DOI:** 10.5334/jbr-btr.1043

**Published:** 2016-03-07

**Authors:** Olivier Ghekiere, Alain Nchimi, Eric Bijnens, Sophie Demanez, Hein Heidbuchel, Paul Dendale

**Affiliations:** 1 Department of Radiology CHC St- Joseph Liège, BE; 2 Department of Radiology Jessa Hospital Hasselt, BE; 3 Faculty of Medicine and Life Sciences, Hasselt University, BE; 4 GIGA cardiovascular disease, Liège University, BE; 5 Department of Cardiology, CCO Liège, BE; 6 Department of Cardiology, Jessa Hospital Hasselt, BE

Sudden cardiac death (SCD) in athletes is an uncommon but dramatic occurrence, with an incidence of 0.6 to 3.6/100,000 per year. SCD is mainly due to malignant ventricular arrhythmias that may occur in case of conduction re-entry abnormality. While hypertrophic cardiomyopathy is the most common cause of SCD in athletes younger than 35 years, coronary artery disease (CAD) is the predominant aetiology in middle age and older athletes. Malignant congenital coronary anomalies are the second most common cause of SCD. Other possible causes of SCD are myocarditis, arrhythmogenic right ventricular cardiomyopathy, dilated cardiomyopathy and less common pathologies – such as myocardial bridging, left ventricular non compaction cardiomyopathy, sarcoidosis and valvular disease. The main morphological and functional conditions predisposing to SCD can be present on the imaging armamentarium.

While echocardiography is the primary imaging modality in athletes with and without clinical symptoms, electrocardiographically (ECG) abnormalities or family history suggesting risk for SCD, cardiac magnetic resonance (CMR) has a growing role for the detection of cardiomyopathies and their differentiation from adaptive athlete’s heart. In addition, CMR provides the unique ability to unveil unsuspected focal or diffuse myocardial fibrosis. Lastly, coronary computed tomography angiography (CCTA) has become a non-invasive modality with high accuracy to exclude obstructive CAD. CCTA is also a highly accurate tool for visualizing myocardial bridging and has been shown to be superior to conventional coronary angiography in delineating the origin and the course of congenital coronary anomalies.

## Competing Interests

The authors declare that they have no competing interests.

